# SAPS 3, APACHE IV or GRACE: which score to choose for acute coronary syndrome patients in intensive care units?

**DOI:** 10.1590/1516-3180.2013.1313474

**Published:** 2013-06-01

**Authors:** Antonio Paulo Nassar, Amilcar Oshiro Mocelin, Fabio Moreira Andrade, Leonardo Brauer, Fabio Poianas Giannini, Andre Luiz Baptiston Nunes, Carlos Augusto Dias

**Affiliations:** I MD. Attending Physician, Intensive Care Unit, Hospital São Camilo, São Paulo, Brazil.; II MD. Local Medical Coordinator, Intensive Care Unit, Hospital São Camilo, São Paulo, Brazil.; III MD. General Medical Coordinator, Intensive Care Unit, Hospital São Camilo, São Paulo. Brazil.

**Keywords:** Acute coronary syndrome, Intensive care units, Prognosis, APACHE, Hospital mortality, Síndrome coronariana aguda, Unidades de terapia intensiva, Prognóstico, APACHE, Mortalidade hospitalar

## Abstract

**CONTEXT AND OBJECTIVE:**

Acute coronary syndromes (ACS) are a common cause of intensive care unit (ICU) admission. Specific prognostic scores have been developed and validated for ACS patients and, among them, GRACE (Global Registry of Acute Coronary Events) has had the best performance. However, intensive care clinicians generally use prognostic scores developed from heterogeneous populations of critically ill patients, such as APACHE IV (Acute Physiologic and Chronic Health Evaluation IV) and SAPS 3 (Simplified Acute Physiology Score 3). The aim of this study was to evaluate and compare the performance of these three scores in a non-selected population of ACS cases.

**DESIGN AND SETTING:**

Retrospective observational study to evaluate three prognostic scores in a population of ACS patients admitted to three general ICUs in private hospitals in São Paulo.

**METHODS:**

All patients with ACS admitted from July 2008 to December 2009 were considered for inclusion in the study. Score calibration and discrimination were evaluated in relation to predicting hospital mortality.

**RESULTS:**

A total of 1065 patients were included. The calibration was appropriate for APACHE IV and GRACE but not for SAPS 3. The discrimination was very good for all scores (area under curve of 0.862 for GRACE, 0.860 for APACHE IV and 0.804 for SAPS 3).

**CONCLUSIONS:**

In this population of ACS patients admitted to ICUs, GRACE and APACHE IV were adequately calibrated, but SAPS 3 was not. All three scores had very good discrimination. GRACE and APACHE IV may be used for predicting mortality risk among ACS patients.

## RESUMO

**CONTEXTO E OBJETIVO:**

Síndromes coronarianas agudas (SCA) são causa comum de admissão à unidade de terapia intensiva (UTI). Escores prognósticos específicos foram desenvolvidos e validados para pacientes com SCA e, dentre esses, o GRACE (Registro Global de Eventos Coronarianos Agudos) tem tido a melhor *performance*. No entanto, os intensivistas normalmente usam escores desenvolvidos para populações heterogêneas de pacientes graves, como o APACHE IV (Avaliação de Saúde Crônica e Fisiologia Aguda IV) e o SAPS 3 (Escore Fisiológico Agudo Simplificado 3). O presente estudo objetiva avaliar e comparar a *performance* desses três escores em uma população não selecionada admitida com diagnóstico de SCA.

**TIPO DE ESTUDO E LOCAL:**

Estudo retrospectivo observacional para a avaliação de três escores prognósticos em uma população admitida com SCA em três UTIs gerais de hospitais particulares em São Paulo.

**MÉTODOS:**

Todos os pacientes admitidos com SCA de julho de 2008 a dezembro de 2009 foram avaliados para inclusão no estudo. Foram avaliadas a calibração e a discriminação dos escores em predizer a mortalidade hospitalar.

**RESULTADOS:**

Um total de 1.065 pacientes foi incluído. A calibração foi adequada para o APACHE IV e para o GRACE, mas não para o SAPS 3. A discriminação foi muito boa para todos os escores (área sob a curva de 0,862; 0,860 e 0,804 para GRACE, APACHE IV e SAPS 3).

**CONCLUSÕES:**

Nesta população de pacientes com SCA admitidos à UTI, os escores GRACE e APACHE IV apresentaram uma calibração adequada, mas o SAPS 3 não. Todos os escores tiveram uma discriminação muito boa. O GRACE e o APACHE IV podem ser usados para predição de mortalidade em pacientes com SCA.

## INTRODUCTION

Acute coronary syndromes (ACS) are a common reason for intensive care unit (ICU) admission.^1^^,^^2^ However, their clinical presentation varies and, therefore, risk stratification is fundamental in order to guide diagnostic and therapeutic approaches.

Many specific prognostic scores have been developed and validated for ACS patients. Among them, the Global Registry of Acute Coronary Events (GRACE)^3^ has had the best performance, probably due to its simple design, given that it does not differentiate between patients with and without ST-elevation ACS, and because it was developed from a large cohort of ACS patients who had not taken part in a clinical trial.

However, intensive care physicians usually use “general” prognostic scores developed from heterogeneous populations of critically ill patients, such as the Acute Physiologic and Chronic Health Evaluation (APACHE)^4^ and Simplified Acute Physiology Score (SAPS).^5^ Although the most-used early versions of these scores, APACHE II^6^ and SAPS II,^7^ did not include ACS patients during their development, their recent versions (APACHE IV^8^ and SAPS 3^9^) did so, and may, theoretically, be used for ACS patients’ risk stratification in general ICUs.

However, before prognostic scores can be widely adopted, they need to be validated, i.e. their performance must be evaluated in a population different from the one on which their development was based.^10^ This validation is accomplished by evaluating the calibration and discrimination of the models. Calibration assesses the degree of correspondence between the estimated probability of hospital mortality and the mortality actually observed for the range of probabilities. The calibration of prognostic scores is usually evaluated by assessing the correlation between predicted and observed mortalities in groups (for example, deciles) of predicted risk. Discrimination assesses the ability of a model to distinguish patients who died from those who survived.^11^

## OBJECTIVE

This study aimed to evaluate and compare the performance of one “specific” ACS prognostic score (GRACE) and two “general” ICU prognostic scores (APACHE IV and SAPS 3) in a Brazilian population of ACS patients admitted to general ICUs.

## METHODS

### Study design and setting

This retrospective cohort study was conducted using data gathered between July 1, 2008, and December 31, 2009, in three Brazilian ICUs. All of these ICUs are medical-surgical and staffed with full-time intensive care specialists, nurses and physiotherapists. During the study period, two ICUs had 31 beds and one had 24. None of them had an explicit ICU admission policy, i.e. patients could come from the emergency department, the operating room, the wards or the catheterization laboratory, or be transferred from other hospitals, at the discretion of the attending physician. Decisions to discharge ACS patients were made by the intensive care specialist and the cardiologist in charge of the patient. During the study period, none of the hospitals had intermediate or coronary care units, and all the patients were discharged to the wards. All three hospitals have specific chest pain protocols in their emergency rooms and catheterization laboratories that are available 24/7. All patients admitted with ST-elevation myocardial infarction are treated with primary angioplasty. Patients admitted with unstable angina and non-ST elevation myocardial infarction are admitted to the ICU and monitored with a continuous electrocardiogram, non-invasive arterial pressure gauge and pulse oximetry. When non-ST elevation ACS patients have refractory chest pain (i.e. pain that does not respond to nitroglycerin, morphine or beta-blocker), acute pulmonary edema or cardiogenic shock, they are immediately transported to the catheterization laboratory as a matter of urgency, after the cardiologist in charge has been contacted. Non-ST elevation ACS patients who remain stable are stratified based on the cardiologist’s decision on the first day after their admission to the ICU. Patients with troponin elevation or dynamic electrocardiographic alterations are usually invasively stratified on the day that follows their admission. All other non-ST elevation ACS patients are non-invasively stratified with stress echocardiography.

### Selection of participants

All consecutively admitted ACS patients aged ³ 18 years in the study period in the three hospitals were included. ACS were defined as typical chest pain with or without electrocardiographic alterations (ST-elevation or depression, T-wave inversion or new left bundle-branch), and with or without troponin elevation. Acute myocardial infarction (AMI) was defined as ACS typical symptom and troponin elevation. Patients who were readmitted during the same hospital stay or who were transferred to another hospital (during their ICU stay or after ICU discharge if still hospitalized) were excluded. The study was approved by the local Ethics Committees and the need for informed consent was waived since no intervention was required and no individual data were expected to be disclosed.

### Data gathering

The data were gathered manually in accordance with the general rules and definitions for the three scores,^3^^,^^8^^,^^9^^,^^12^ using a specific form to be filled out by the intensive care specialist on duty at the time of the patient’s admission (appendix). The data to be entered on the form included demographic information (age and gender), patient origin (emergency room, ward or transfer from another hospital) and diagnosis (unstable angina, non-ST elevation ACS or ST elevation ACS). Patients admitted from the catheterization laboratory after primary angioplasty were considered to have originated from the emergency room. Since the SAPS 3 and GRACE variables were collected within the first hour after admission, they were entered on the form by the intensive care specialist who admitted the patient. APACHE IV diagnostic data were gathered upon admission. Other APACHE IV variables were gathered within 24 hours of admission, from the medical records, by a nursing student trained in severity scores or by the local ICU medical coordinator. None of the physicians who inserted data in the forms were involved in the data analysis. All the data were entered into a Microsoft Excel^®^ spreadsheet, which was used to estimate in-hospital mortality risks. For SAPS 3, the global equation was utilized.

### Statistical analysis

Statistical analyses were performed using the Statistical Package for the Social Sciences (SPSS), version 10.0, and MedCalc version 9.0 softwares. Continuous variables were presented as the median and interquartile range (IQR). Categorical variables were presented as absolute values and percentages.

The prognostic performance of the different scores was evaluated in terms of calibration and discrimination. The calibration was assessed using the Hosmer-Lemeshow goodness-of-fit test C-statistic, which evaluates the agreement between the observed and expected numbers of survivors and non-survivors across all of the strata of probabilities of death.^13^ A high P value (P > 0.05) indicates a good fit for the model. Standardized mortality ratios (SMRs) with their respective 95% confidence intervals (CI) were calculated by dividing observed by predicted rates.

The score discrimination was assessed by calculating the area under the receiver operating characteristic curve (AUROC) and its 95% CI. The discrimination was considered to be excellent, very good, good, moderate and poor with AUROC values of 0.9-0.99, 0.8-0.89, 0.7-0.79, 0.6-0.69 and < 0.6, respectively. Pairwise comparisons of the AUROCs were performed using the De-Long method.^14^

## RESULTS

During the study period, 1229 patients were admitted with a diagnosis of ACS. A total of 164 patients were excluded due to incomplete data that prevented calculation of one or more scores (n = 93), readmission during the same hospital stay (n = 65) or transfer to another hospital (n = 6). The characteristics of the patients included are displayed in Table 1. In-hospital mortality was 2.4%.

**Table 1.** Baseline patient characteristics and main outcomes

Characteristics	
Age [years; median (IQR)]	61 (52.25-73)
Male [n (%)]	635 (59.6)
Location before ICU admission [n (%)]	
Emergency room	876 (82.3)
Ward	43 (4.0)
Other hospital	146 (13.7)
Reason for admission [n (%)]	
UA/NSTEMI	915 (85.9)
STEMI	150 (14.1)
ICU LOS [days; median (IQR)]	2.30 (1.61-3.50)
In-hospital mortality [n (%)]	26 (2.4)

IQR = interquartile range; ICU = intensive care unit; UA = unstable angina; NSTEMI = non-ST-segment elevation myocardial infarction; STEMI = ST-segment elevation myocardial infarction; LOS = length of stay.

**Figure 1.**
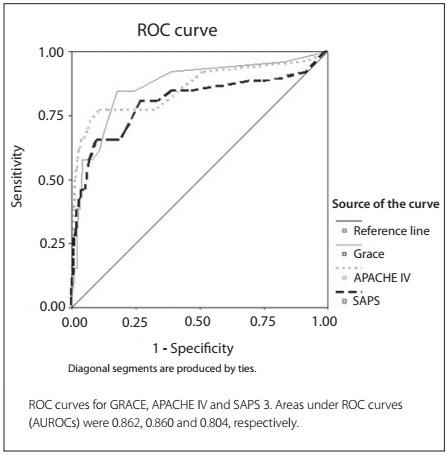
Receiver operating characteristic curves (ROC curves).

GRACE and APACHE IV presented appropriate calibration, but SAPS 3 did not. SAPS 3 overestimated in-hospital mortality (Table 2). The discrimination was very good for all scores (Figure 1). The AUROC was 0.862 (95% CI: 0.840-0.883) for GRACE; 0.860 (95% CI: 0.838-0.880) for APACHE IV; and 0.804 (95% CI: 0.779-0.828) for SAPS 3. There were no differences in discrimination among the scores (GRACE versus APACHE IV, P = 0.955; GRACE versus SAPS 3, P = 0.282; APACHE IV versus SAPS 3, P = 0.135).

**Table 2.** Performance of the scores

SCORE	Predicted mortality	SMR (95% CI)	H-L statistic	P value
GRACE	3.1%	0.77 (0.47-1.08)	11.0	0.25
APACHE IV	3.2%	0.77 (0.46-1.07)	12.5	0.27
SAPS 3	7.9%	0.31 (0.11-0.50)	51.8	< 0.001

SMR = standardized mortality ratio; CI = confidence interval; H-L = Hosmer-Lemeshow statistic for goodness-of-fit. GRACE = Global Registry of Acute Coronary Events; APACHE IV = Acute Physiologic and Chronic Health Evaluation IV; SAPS 3 = Simplified Acute Physiology Score 3.

## DISCUSSION

In this study, one “general” score (APACHE IV) and one “specific” ACS prognostic score (GRACE) showed appropriate calibration and very good discrimination among a non-selected ACS population admitted to ICUs. Another “general” score, SAPS 3, which included ACS patients in its development, also showed very good discrimination, but poor calibration, with overestimation of in-hospital mortality.

Interest in using prognostic scores originates from the fact that a very heterogeneous population of critically ill patients is admitted to ICUs. Because of this, stratification of patients’ risk could theoretically enable better resource allocation and better comparison of ICU performance over certain periods and between different ICUs. ACS is a common reason for ICU admission, and specific scores have also been developed for ACS. These scores have showed good accuracy with regard to ACS risk stratification,^15^^,^^16^^,^^17^^,^^18^ and risk stratification is recommended in the ACS treatment guidelines in order to guide therapeutics.^19^ Among these scores, GRACE seems to have the best performance. ^15^^,^^16^^,^^17^^,^^18^ This was developed in a large cohort of ACS patients from many countries who had not taken part in clinical trials. Moreover, it included a few variables that are usually measured upon ACS patients’ admission.^3^ Therefore, GRACE seems to be an appropriate prognostic score for risk stratification in the “real world”, and this is the reason why it was the one chosen for the present study.

On the other hand, because of the variability of conditions responsible for ICU admissions, healthcare workers have used “general” prognostic scores developed in very heterogeneous populations of critically ill patients. Among these, APACHE and SAPS have been the scores most used in clinical practice and in research. However, except for APACHE III,^20^ the older versions of the scores were not developed in cohorts with ACS patients, although they were validated in this group of patients afterwards.^2^^,^^21^^,^^22^ ACS patients were included in developing the newest versions (APACHE IV and SAPS 3), but these still have not been validated in an external ACS population.

To the best of our knowledge, only one previous study compared “general” and “specific” prognostic scores for risk stratification in AMI cases. That study compared seven scores, among which the one most used in studies was APACHE II, and did not find any substantial differences in accuracy between most of them.^23^ A Spanish group has periodically evaluated “general” scores among ACS patients admitted to ICUs or coronary units. In an initial study, the group observed that APACHE III presented greater accuracy than SAPS II.^2^ Subsequently, a comparison between APACHE II, SAPS II and MPM-II (mortality probability models) showed that all of these scores presented appropriate calibration and good discrimination for AMI patients.^22^ Recently, the same group found that a prognostic model that included the Killip classification and APACHE II showed good accuracy.^24^

Our study was the first one to compare the performance of two recent general scores (APACHE IV and SAPS 3) and one specific ACS score (GRACE). SAPS 3 demonstrated poor calibration, which may have been due to inappropriateness for assessing an ACS population or to different clinical presentation and treatment choices in this specific population.^25^^,^^26^ Nevertheless, some studies have already suggested that SAPS 3 underestimates in-hospital mortality among general critical care patients, which may be due to the model itself.^27^^,^^28^

On the other hand, APACHE IV and GRACE presented appropriate calibration and similar accuracy. Choosing between these scores depends on the aim. If the aim is to study ACS patients in ICUs or to evaluate health clinic performance in relation to ACS cases, GRACE would be the best choice. GRACE is a simpler score that only includes seven variables: these are gathered on admission and have prognostic implications for ACS patients, such as electrocardiographic alterations, elevation of cardiac biomarkers and Killip classification. If the purpose is to compare ACS patients against patients admitted with other diagnoses or to evaluate all patients admitted to the ICU, APACHE IV is a better option. However, completion of data input depends on data gathered within the first 24h after ICU admission, requires greater effort and precludes its use as a risk stratification tool to guide initial therapy. Hence, we believe that choosing one score does not exclude the other, but using both could provide a broader overview of ACS patients.

Our study has potential limitations. First, although it was designed as a multicenter study, all three ICUs observed are located in the same city and provide similar standards of care. Therefore, our cohort may not be representative of a larger population. Two points that provide examples of this limitation were the percentage of non-ST elevation ACS patients, which was larger than in the original GRACE cohort,^3^ although similar to a Brazilian registry,^29^ and the observation that all ST-elevation myocardial infarction patients were treated with primary angioplasty, which is only routinely performed in reference centers.^29^ Second, we did not gather some data that could be important for prognostic information, such as the use of medications like aspirin, clopidogrel, beta-blockers and statins, because these data were not available in our database. These data could give better insight regarding our patients’ profile and the healthcare that they received. However, since our sole objective was to evaluate the calibration and discrimination of three prognostic scores in a specific population, we believe that the absence of these data did not have any impact on our results.

## CONCLUSIONS

Our study showed that APACHE IV and GRACE had appropriate calibration and very good discrimination in a non-selected population of ACS cases admitted to general Brazilian ICUs. Even though SAPS 3 was developed based on a very large sample from different countries, it was not properly calibrated for this population, although its discrimination was similar to that of the other scores. We recommend that APACHE IV and GRACE should be chosen for mortality risk prediction for ACS patients admitted to healthcare facilities with profiles similar to ours.
